# Cellulolytic Potential of Newly Isolated Alcohol-Tolerant *Bacillus methylotrophicus*

**DOI:** 10.3390/ma18143256

**Published:** 2025-07-10

**Authors:** Anna Choińska-Pulit, Justyna Sobolczyk-Bednarek, Wojciech Łaba

**Affiliations:** 1 “Poltegor-Institute”, Opencast Mining Institute, Parkowa 25, 51-616 Wrocław, Poland; anna.choinska-pulit@igo.wroc.pl (A.C.-P.); justyna.sobolczyk@igo.wroc.pl (J.S.-B.); 2 Department of Biotechnology and Food Microbiology, Faculty of Biotechnology and Food Science, Wrocław University of Environmental and Life Sciences, Chełmońskiego 37, 51-630 Wrocław, Poland

**Keywords:** cellulase production, cellulose hydrolysis, optimization, *Bacillus methylotrophicus*

## Abstract

Reprocessing lignocellulosic waste to obtain new products for industrial purposes is a vital part of circular economy. This paper reports the cellulase production by newly isolated *Bacillus methylotrophicus* cultured on lignocellulosic agro-industrial by-products, out of which brewer’s spent grain (BSG) was selected as most beneficial. Plackett–Burman design was used for screening medium components, while Box–Behnken design was further applied to model the impact of the three most influential variables. The maximum approximated cellulase activity was 0.469 U/mL (1 U = 1 µmol of reducing sugars/1 min), at 48.6 g/L substrate, 5.3 g/L ammonium sulfate, pH 6.1. The partially purified cellulase was characterized, which demonstrated broad range of optimal pH (6.5–9.4), temperature (50–60 °C), and sensitivity to metals. Changes in lignin and pentosans content was demonstrated as a result of BSG hydrolysis with a cell-free cellulase preparation. The produced enzyme was used for hydrolysis of various chemically pretreated (NaOH and H_2_SO_4_) cellulosic substrates, where for reused alkali-pretreated BSG (after microbial enzyme production) the saccharification efficiency was at a level of 25%. The cellulolytic potential of the bacterial strain, along with its resistance to ethanol, present a beneficial combination, potentially applicable to aid saccharification of lignocellulosic by-products for biofuel production.

## 1. Introduction

Operations in food industry and agriculture generate large volumes of residues every year. One of the major by-products in the brewing industry, the management of which is technologically and economically challenging, is brewer’s spent grain (BSG). Each 100 L of regional beer produces from 20 to 22 kg of BSG [1]. Its accumulation also poses a significant ecological problem. Therefore, there is a growing trend to seek novel applications that will change the traditional approach to waste products and exploit them as raw materials and value-added compounds while minimizing the waste stream [2,3]. Lignocellulosic waste, including BSG, can serve as an example because it can be considered as a substrate for alcoholic fermentation to produce a valuable product—bioethanol.

Over the past two decades, the interest in bioethanol, which is an alternative source of energy for fossil fuels, has increased. Conventionally, this alcohol is produced by processing starch- and sucrose-based raw materials through enzymatic liquefaction and saccharification, yielding a relatively pure glucose pool. However, the crisis related to food and feed production caused the need to produce ethanol from other sources. For this reason, there is a growing interest in the use of lignocellulose waste like BSG for sustainable bioethanol production. The processing of agricultural waste into ethanol follows a general procedure that involves three main operations: raw material pretreatment–delignification, which is necessary to release cellulose and hemicellulose prior to hydrolysis; hydrolysis of cellulose and hemicellulose to form fermentable sugars (glucose, xylose, arabinose, galactose, and mannose); and fermentation of the resultant sugars into ethanol [4]. The only problem is that technologies are necessary for an ecological and cost-effective extraction of sugars from the complex lignocellulose network, because the cellulose fraction in lignocellulose waste is not easily accessible to the enzyme digestion required for ethanol production [5]. The use of physical and chemical treatments can overcome this limitation by increasing the efficiency of enzymatic hydrolysis. Chemical hydrolysis of cellulose with the use of strong acids allows for obtaining high sugar yields without the need to use elevated process temperatures. Despite the high efficiency of acid hydrolysis, this process requires the use of acid-resistant reactors and generates large amounts of corrosive waste. The solution may be to use more diluted acids, which also increases the cost-effectiveness of the process [6,7].

Bacteria of the genus *Bacillus* are widely distributed in the natural environment and many of them are species safe for humans and animals (the GRAS status, Generally Recognized as Safe). Representatives of the genus *Bacillus* are characterized by high growth rate, the ability to produce endospores, and an efficient system for the biosynthesis and secretion of extracellular enzymes allowing for efficient product synthesis reaching a level of 20–25 g/L (Deb et al., 2013) [8]. These are the reasons why many *Bacillus* strains are used in the production of commercial preparations, including enzymes, biosurfactants, antibiotics, insecticides, and vitamins, along with other secondary metabolites [9,10,11]. It is estimated that half of the currently available biopreparations are obtained from this group of microorganisms [12]. Among the enzyme preparations produced by species of the genus *Bacillus*, cellulases, used in the production of bioethanol, deserve special attention. The cellulosic enzyme system contains at least three main types of enzymes, including endo-1,4-beta-D glucanase (EC 3.2.1.4), exo-1,4-beta-D glucanase (EC 3.2.1.91), and beta-glucosidase (EC 3.2.1.21) [13]. However, the high costs of cellulase preparations and limited specificity of cellulases forces the use of large amounts of enzymes to satisfy the amount of hydrolysable cellulose [14,15]. To obtain the maximum production of cellulase enzyme, this problem can be addressed by considering cellulase production kinetics and optimizing the parameters for production operating conditions [14,16]. Lignin inhibits cellulolytic enzymes by forming a physical barrier that limits enzyme access to cellulose and by adsorbing the enzymes, thereby reducing enzymatic activity [17,18]. This process depends on the type of biomass and the pretreatment or isolation method used [7,17,18,19,20]. Physical parameters of the hydrolysis process, such as temperature, pH, agitation, and the chemical composition of the culture medium, play an important role in the cellulase production efficiency of microorganisms. Therefore, thorough optimization of culture conditions is necessary to increase the enzyme yield.

Another limitation in bioethanol bioproduction is that the toxicity of alcohol compounds slows or inhibits cell growth, reducing production efficiency. *Saccharomyces cerevisiae* yeast is a natural and widely used producer of ethanol, while bacteria produce lower amounts of alcohol but offer advantages such as rapid growth, the ability to utilize diverse carbon sources, and the availability of genetic and molecular tools for their modification [21]. Bacterial alcohol tolerance is generally lower than that of yeast, making alcohol toxicity a more significant challenge. One strategy to address this is the development of strains with enhanced tolerance to these compounds [21]. Among bacteria of the genus *Bacillus*, some facultatively anaerobic species, such as *Bacillus cereus*, can be utilized for bioethanol production due to their beneficial traits [7].

The aim of this study was to optimize the parameters for cellulase production by a newly isolated alcohol-tolerant *Bacillus methylotrophicus* strain in BGS-based medium, and to characterize the enzyme in terms of its applicability in lignocellulosic biomass saccharification.

## 2. Materials and Methods

### 2.1. Isolation and Molecular Identification of the Bacterial Strain

The tested bacterial strain was isolated from a 70% ethanol storage tank. It demonstrated exceptional resistance to high ethanol concentrations and exhibited, among other traits, significant cellulolytic activity.

Identification of the isolated strain was performed based on the sequence homology of the 16S rDNA. The PCR reaction was performed with universal primers: (27 F) AGAGTTTGATCGTGGCTCAG and (1492 R) GGTTACCTTGTTACGACTT under standard procedure. The PCR product was sequenced using the same primers and the obtained sequence was subjected to Ribosomal Database Project (RDP) release 11 [22] to acquire related 16S rDNA sequences. Sequence alignment and cladogram construction were performed with MAFFT version 7 [23].

### 2.2. Evaluation of Alcohol Tolerance

The resistance of the selected bacterial strain to ethanol and methanol was tested. The experiment was carried out in test tubes containing 15 mL LB medium with the addition of alcohol at the following concentrations: 0% (control), 2%, 5%, 10%, 20%, 50%, 60%, and 70%, inoculated with bacterial culture (1.0 × 10^7^ cfu/mL). Cultures were incubated at 30 °C for 24 h in a rotary shaker (130 rpm/min). Bacterial growth was assessed by optical density (OD) measurement at 600 nm wavelength after cultivation. Total count of bacterial population was measured by the plate method on Luria–Bertani (LB) agar (results expressed in colony forming units per 1 mL culture [cfu/mL] after 24 h incubation at 30 °C). All measurements were performed in triplicate.

### 2.3. Bacterial Culture in CMC Medium

The tested strain was cultured in 100 mL of medium containing ([g/L]) CMC-Na (carboxymethylcellulose sodium salt), 10.0; YE (yeast extract), 5.0; peptone, 5.0; K_2_HPO_4_, 1.0; MgSO_4_·7H_2_O, 0.2; and NaCl, 1.0. The agitated culture was carried out at 30 °C, for 72 h at 180 r.p.m. Samples of culture fluids were withdrawn and centrifuged (4500 r.p.m., 12 min, 4 °C), and then pH and cellulolytic activity were determined in the supernatants. Overnight culture in LB medium at 30 °C, followed by centrifugation (10 min, 7000× *g*, 4 °C) and suspension of cells in sterile distilled water, served as the inoculum. Approximately 4·× 10^8^ cells per 100 mL of culture medium were used, standardized by OD600 measurement.

### 2.4. Selection of Lignocellulosic Substrate

Selection of the lignocellulosic residue as a substrate for protease production was performed. Submerged bacterial cultures were carried out in a media containing 2% of the substrates in deionized water (pH 6.5): brewers spent grain (BSG), beet pulp, apple pomace, and wheat bran. Media were inoculated as described above. Cultures were incubated at 30 °C for 4 days. Cellulase activity was tested in the culture fluid supernatant at 24 h intervals.

### 2.5. Determination of Cellulolytic Activity

The cellulase assay was performed by a modified method of Xiao et al. [24]. The reaction mixture contained 30 μL of 1% CMC (carboxymethyl cellulose sodium salt), 20 μL of phosphate buffer pH 6.5, and 10 μL of enzyme solution. After 30 min of incubation at 50 °C, the reaction was terminated with 60 μL of 3,5-dinitrosalicylic acid (DNS). The mixture was heated for 5 min at 95 °C for color development, cooled, and diluted with 480 μL of deionized water. The absorbance was measured at 530 nm wavelength in 10 mm light-path microcuvettes. One unit (1 U) of cellulolytic activity was defined as the amount of enzyme that released 1 µmol of reducing sugars (glucose) during 1 min.

### 2.6. Screening of Process Parameters

Preliminary screening of independent variables, comprising six medium components and pH, was performed according to a two-level Plackett–Burman design. The aim was to identify the most important factors that impact the production of cellulase in submerged cultures of the isolate grown on BSG as the substrate, according to the experimental layout presented in the Table S1. Based on linear regression coefficients and their statistical significance, the effect of each input variable was verified.

### 2.7. Optimization of Culture Medium for Cellulase Production

Three independent variables, i.e., the concentration of substrate (BSG) (X_1_), concentration of ammonium sulfate (X_2_), and pH of the culture medium (X_3_), were modeled using the Box–Behnken experimental design, in order to describe their influence on the production of cellulase by the tested bacterial strain and to establish the optimal composition of the medium. The experimental layout comprised 15 runs, including 3 replications of the central point, according to Table 1. Finally, the relationship between the input variables and the response (Y) was expressed in the form of the following polynomial equation, after establishing regression coefficients:

Y = β_0_ + β_1_X_1_ + β_2_X_2_ + β_3_X_3_ + β_11_X_1_X_1_ + β_22_X_2_X_2_ + β_33_X_3_X_3_ + β_12_X_1_X_2_ + β_13_X_1_X_3_ + β_23_X_2_X_3_ (1)

including the intercept (β_0_), linear coefficients (β_1_, β_2_, β_3_), quadratic coefficients (β_11_, β_22_, β_33_), and interaction terms (β_12_, β_13_, β_23_). The analysis of the model, response surface plots, and ANOVA statistics were performed with Statistica 14 software (TIBCO Software Inc., Palo Alto, CA, USA).

### 2.8. Enzymatic Hydrolysis of Lignocellulose

Three agro-industrial residues were subjected to enzymatic hydrolysis with the enzymatic preparation produced using the *B. methylotrophicus* strain under optimized culture conditions. Raw culture fluid after centrifugation (12 k r.p.m., 10 min, 4 °C) was concentrated by ultrafiltration using the Labscale TFF System (Millipore, Bedford, MA, USA) equipped with the Pellicon XL 50 Cassette (Biomax, 8 kDa cut-off, Merck KgaA, Darmstadt, Germany). The resultant preparation served as the catalyst for enzymatic hydrolysis of lignocellulose.

Milled (<1 mm) lignocellulosic residues, i.e., BSG (raw and as a residue from enzyme production), wheat straw, oat straw, and corn straw, were initially pretreated using either alkaline (4% NaOH) or acidic (4% H_2_SO_4_) thermal treatment in an autoclave (121 °C, 1 h), according to the protocol described by Lemões et al. [25]. Reaction mixtures contained substrate 2% (d.m./v), enzyme 10 U, and acetic buffer 0.05 M, at pH 5.4 and 50 mL total volume. The reaction was run for 48 h at 50 °C. After pretreatment, the solid fraction was separated from the liquid fraction and was washed with water to remove the excess alkali/acid until the pH was 4.5–5.0. It was then dried at 45°C. Withdrawn samples were cooled and subsequently the content of reducing sugars was determined using the DNS method. The degree of saccharification was measured as the amount of sugar released due to hydrolysis, by subtracting the carbohydrates present prior to the addition of the enzyme. The result was expressed as a percentage of reducing sugars relative to the initial content of the cellulosic substrate.

### 2.9. Purification of Cellulase

The clarified and concentrated culture fluid was subjected to enzyme purification with column chromatography on the BioLogic DuoFlow system (BioRad, Hercules, CA, USA). During the first stage, ion exchange chromatography (IEC) was performed with the HiTrap DEAE Fast Flow (Cytiva, Marlborough, MA, USA), 10 mL, in 0.05 M Tris-HCl buffer pH 9.0 and 2.0 mL/min flow rate. Elution gradient up to 0.7 M NaCl was used. Pooled fractions with cellulolytic activity were further purified with a HiPrep 26/60 Sephacryl S-200 HR gel filtration column (Cytiva, USA) in 0.05 M Tris-HCl buffer pH 9.0 containing 10 mM NaCl, at 1.0 mL/min flow rate. Active fractions were pooled, concentrated, and used for cellulase characterization.

### 2.10. Characterization of Cellulase

The effect of temperature on the cellulase activity was tested in the range 35–70 °C, with a 5 °C interval, at pH 7.0 in 0.05 M phosphate buffer. The effect of pH of the reaction mixture on the cellulase activity was tested at 50 °C in 0.05 M phosphate buffer pH 5.1–7.7 and 0.05 M tris-HCl buffer pH 8.3–9.9. The optima were precisely specified by determining maxima on the quadratic regression equations applied to the experimental data.

The effect of several compounds was tested for their impact on the cellulase enzyme. Salts, i.e., ZnSO_4_, FeSO_4_, MgSO_4_, CuSO_4_, CaCl_2_, MnSO_4_, and metal chelator EDTA were used at 5 mM concentration; non-ionic surfactants, Tween 80, Triton X-100, and Brij 35, were used at 1% (*w*/*v*) concentration. After mixing with an enzymatic solution, cellulase activity assay was performed at standard conditions (50 °C, pH 7.0).

### 2.11. Determination of the Chemical Composition of the Waste Material

The determinations were carried out by the Łukasiewicz Research Network—Poznań Institute of Technology. The scope of research included determination of cellulose, lignin, and hemicelluloses (substances soluble in 1% aqueous NaOH). Preparation of the sample for cellulose, lignin, and soluble substances in 1% NaOH consisted of its comminution in a Pulverisette 15 cutting mill (Fritsch, Idar-Oberstein, Germany) and sieving the particles through 0.5 mm and 1.0 mm mesh in order to obtain the analytical fraction with suitable graining, for subsequent analysis of the chemical composition, based on established research methods [26,27,28].

The following determinations were performed: water content (humidity) by the drying–weighing method at 105 ± 2 °C [26]; content of extractive substances soluble in ethanol using the Soxhlet apparatus (PN-P-50092: 1992); the content of substances soluble in 1% NaOH, which primarily indicates the amount of hemicelluloses present in the lignocellulosic material, although some amounts of cellulose and lignin also pass into the extract; cellulose content by the Seifert method [26]; and Klason lignin content (insoluble in acid) by TAPPI [26,27,28].

### 2.12. SEM Analysis

A Hitachi S3400 scanning electron microscope (Tokyo, Japan) (total magnification ×4000) was used to observe raw, enzymatic, and microbiologically treated BSG.

## 3. Results and Discussion

### 3.1. Identification of the Bacterial Isolate

**Figure 1:**
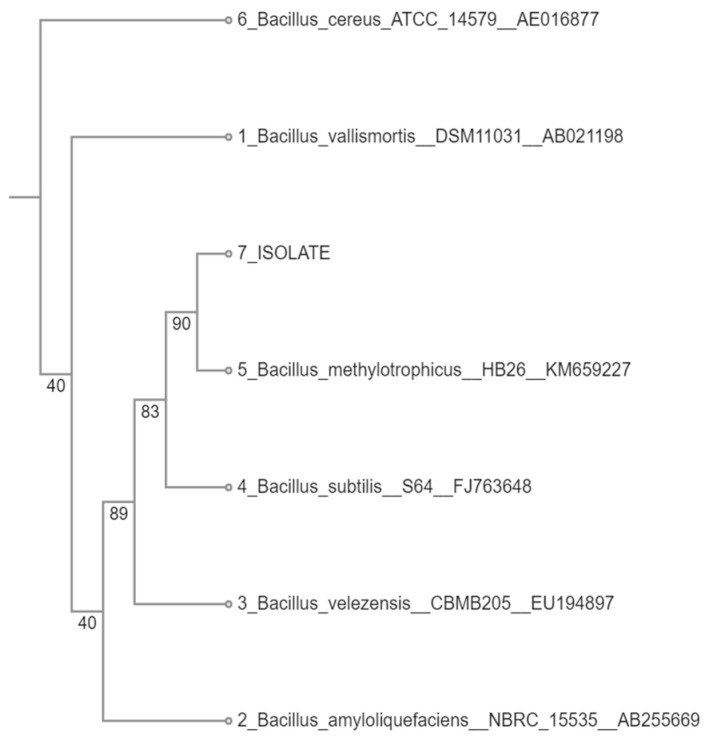
Cladogram built with the neighbor-joining method based on the homology of 16S rDNA. Bootstrap values are indicated at the branching points (out of 100 replicates of bootstrap sampling). *Bacillus cereus* ATCC 14579 was used as an outgroup.

The partial sequence of the 16S rDNA with 1421 nucleotide span was obtained for the tested bacterial isolate. Based on the homology with corresponding sequences of type and non-type strains retrieved from the RDP database, the highest similarity was determined toward both *B. methylotrophicus* and *B. subtilis* with the maximum 0.997 sequence match score calculated with the SeqMatch tool. The constructed cladogram placed the tested isolate in the same clade as *Bacillus methylotrophicus*, confirming it as the closest related species (Figure 1). One must consider that taxonomic differentiation between *B. methylotrophicus*, *B. amyloliquefaciens* subsp. *plantarum*, *B. velezensis*, and *B. oryzicola* has been a baffling issue. Based on the phylogenetic analysis of the core genome of those closely related species, Dunlap et al. [29] suggested that the *B. methylotrophicus*, *B. amyloliquefaciens* subsp. *plantarum*, and *B. oryzicola* species should be considered later heterotypic synonyms of *B. velezensis*. Additionally, based on 16S rDNA sequence homology, *Bacillus methylotrophicus* was identified as the closest relative to *B. velezensis*, a finding further supported by Fan et al. [30]. This appears to be consistent with the results of our study. The available literature data describing this species as a biocontrol agent indicate its ability to promote plant growth [30,31,32]. However, several recent studies also mention the cellulolytic capabilities of this bacterial species [33,34,35].

### 3.2. Alcohol Tolerance of the Tested Isolate

The measurements of the strain’s alcohol tolerance showed that the strain tolerated high ethanol concentrations after 24 h exposition where, at concentrations of methanol and ethanol in a range of 10–20%, a decrease in abundance by two logarithmic orders was observed (1·× 10^5^ cfu/mL), and at concentrations of both alcohols in the range of 40–60%, by 5 logarithmic orders (3·× 10^2^ cfu/mL) in relation to the control sample without alcohol added (1·× 10^7^ cfu/mL, OD_600_ = 0.50 ± 0.04). Bacterial counts for ethanol concentrations 60% and 70% reached the lower limit of detection (below 2·× 10^2^ cfu/mL), but viable bacteria were present up to 70% ethanol (in the culture fluid, the optical density OD_600_ = 0.00 relative to the sterile control medium). At the level of 5% alcohol content, the maximum number of microorganisms was recorded in the amounts of 4.20·× 10^7^ and 3.86·× 10^7^ cfu/mL for ethyl and methyl alcohols, respectively. Starting at a concentration of 40%, a gradual decline in microbial count was observed. The *B. methylotrophicus* strain proved to be more resistant to methanol, maintaining the count at the level of 2.13·× 10^2^ cfu/mL in 70% alcohol solution. Ezebuiro et al. [4] and Abood et al. [36] described alcohol-tolerant *Bacillus* strains in their studies, which exhibited tolerance to ethanol concentrations of 6% and 5% (*v*/*v*), respectively, along with high cellulolytic activity, a feature advantageous in terms of bioethanol production. Bacterial alcohol tolerance is typically lower than that of yeast. As a consequence, alcohol toxicity is a significant limitation in bioethanol production by bacterial strains. This aspect is so significant that OMIC technologies are currently being developed to overcome this problem [21].

### 3.3. Cellulolytic Capability of the Isolate

**Figure 2:**
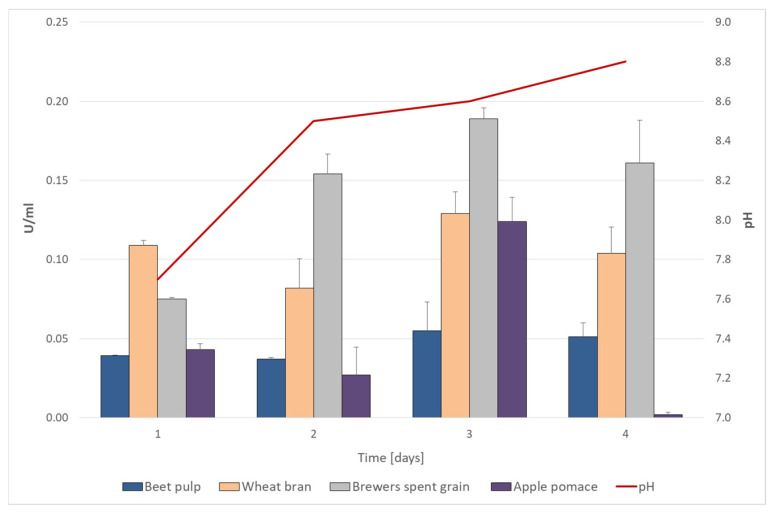
Cellulolytic activity and pH (for BSG) of the *B. methylotrophicus* strain in culture with 2% addition of four selected lignocellulosic substrates: beet pulp, wheat bran, spent grain, and apple pomace.

The activity of cellulases produced on four different lignocellulosic residues by the *B. methylotrophicus* strain was compared. Significantly higher cellulase activity was observed in the culture on BSG (0.19 U/mL), followed by the wheat bran (0.13 U/mL) and apple pomace (0.12 U/mL) (Figure 2). As a consequence, BSG was selected for further research. In all tested cultivation variants, the cellulase activity reached its maximum on the third day of the experiment, during the early stationary growth phase. During cultivation, the medium with BSG was gradually alkalinized, due to the hydrolysis and metabolizing proteins contained in the medium from pH = 7.7 to pH = 8.8 (Figure 2).

A similar level of cellulolytic activity (0.161 U) obtained in the culture of *B. amyloliquefaciens* SS35 grown on CMC prior to optimization was reported by Singh et al. [37]. Likewise, Li and Yu [38] reported similar growth kinetics and extracellular cellulase production by *Bacillus* sp. L1 cultured in complex medium broth. In these studies the maximum cellulase activity occurred during the post-stationary phase (48 h), but no cellulase activity was detected during the early exponential growth phase.

### 3.4. Screening of Process Parameters with the Plackett–Burman Design

**Figure 3:**
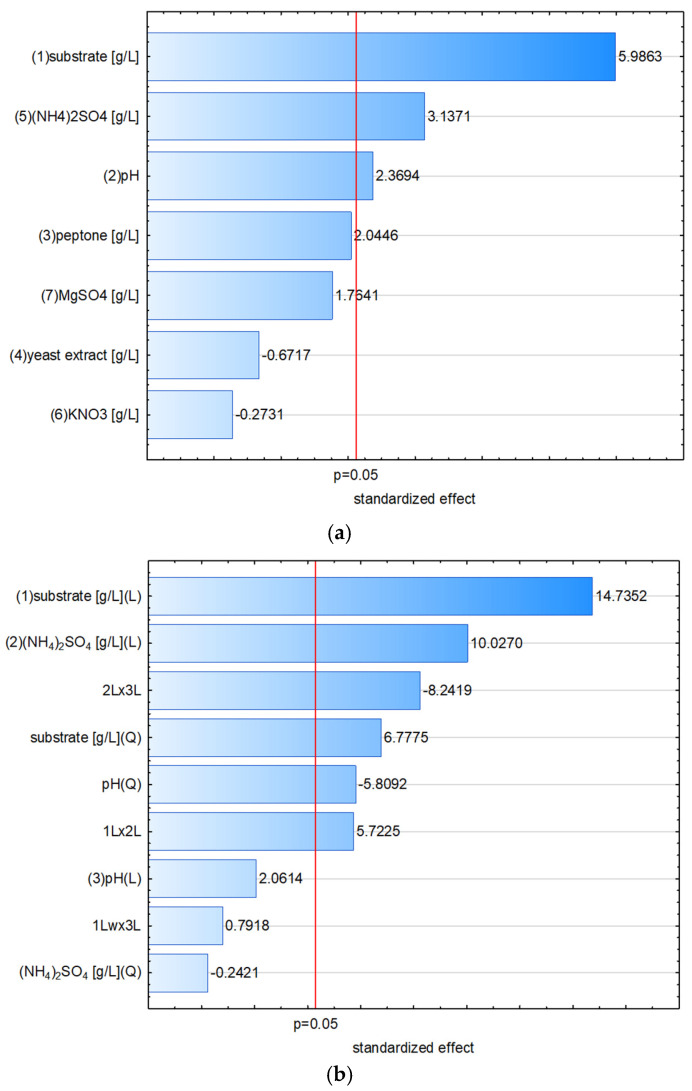
Pareto chart of standardized effects from the (**a**) Plackett–Burman design (linear effects) and the (**b**) Box–Behnken design, prior to model reduction (linear—L, quadratic—Q, and interactive linear—L × L effects).

The Plackett–Burman screening design serves as a tool for the selection of multiple independent variables that affect the measured response, based on the statistical significance of linear regression coefficients. In this study, it was applied to analyze the impact of seven components of the culture medium, to maximize the cellulase production by the tested bacterial train. According to the Pareto chart of effects, the dominant effect of the substrate concentration in culture media on the activity of cellulases was confirmed (Figure 3a).

A higher cellulolytic activity was observed in the presence of 4 g/L of substrate compared to the content of 1.5 g/L. The presence of an additional nitrogen source in the form of ammonium sulfate and the initial pH of the medium were the other factors that significantly influenced the biosynthesis of cellulases by the tested bacteria. The addition of 1 g/L ammonium sulfate increased cellulase activity compared to the medium without it, while pH 7 was more favorable than pH 6. The addition of other compounds like MgSO_4_ or supplementary nitrogen sources, i.e., peptone, yeast extract, and KNO_3_, did not impose a statistically significant effect on the cellulase production.

The selection of culture medium components for the production of cellulases by bacteria from the genus *Bacillus*, besides the main substrate, typically incorporate additional sources of carbon, nitrogen, or mineral compounds. Numerous studies incorporated CMC as the main source of carbon and an inducer, at concentrations varying from 2 g/L to 20 g/L [39]. In some cases, pure carbohydrates like glucose, lactose, and maltose are a reasonable choice [40,41]. Nevertheless, the current trend is to apply agro-industrial by-products to facilitate their subsequent valorization and to minimize the enzyme production cost. Obviously, the optimal content of the substrate needs to be established.

Among supplementary nitrogen sources commonly used for culturing cellulase-producing bacilli, either organic sources like yeast extract and peptone or inorganic ones such as ammonium chloride, ammonium sulfate, and sodium nitrate are typically used. For instance, the most effective production of cellulase by *B. licheniformis* KY962963 was achieved in the presence of glucose and peptone, when grown on CMC [42]. *Bacillus albus* MN755587, however, effectively produced cellulase in the medium supplemented with yeast extract, regardless of the synthetic carbon source—either CMC, glucose, maltose, or lactose [41]. Likewise, peptone and yeast extract were the most influential additives in cultures of cellulolytic *B. cereus* grown on poplar twigs [43]. In some cases natural lignocellulosic compounds prove to be more applicable nutrient sources than pure sugars. Efficient yield of cellulase in the culture of *B. pseudomycoides* occurred during growth on sugarcane bagasse, supplemented with yeast extract [40].

In our study, by contrast, none of the organic nitrogen sources exerted a significant impact on cellulase production by *B. methylotrophicus*. However, the process was positively affected by the presence of ammonium sulfate. Hence, the concentration of the main substrate, the concentration of ammonium sulfate, and the initial pH of the medium were selected as the variables to be optimized using multiple non-linear regression methodology.

### 3.5. Optimization of Cellulase Production with Box–Behnken Design

Considering the results of the Plackett–Burman design, variables such as concentrations of the substrate and ammonium sulfate and the medium pH were further modeled using a Box–Behnken design (Table 1). The Pareto chart illustrates the degree to which individual components of the model affect the dependent variable, i.e., the level of cellulolytic activity obtained in *B. methylotrophicus* cultures (Figure 3b). It was found that both the substrate content in the culture medium and the ammonium sulfate exerted the greatest impact on the production of cellulases, followed by a less notable, but statistically significant effect of the medium pH. The dominant influence of the substrate content was non-linear, while only a linear relationship was found in the case of ammonium sulfate. The minor effect of pH was of a non-linear nature. Nevertheless, significant interactions of substrate vs. ammonium sulfate and pH vs. ammonium sulfate produced a more complex overall picture. The analysis of variance confirmed the significance of these components in the model. The original model was reduced by removing insignificant terms, and ANOVA was performed to evaluate the statistical significance of the remaining factors and to verify the adequacy of the reduced model (Table 2). The coefficient of determination R^2^ = 0.8999 suggests that the model described nearly 90% of the variability in the response, in relation to the values of input variables. In addition, the statistically insignificant “lack-of-fit” test demonstrated the correct relationship of pure error to the variability within residuals.

The relationships established in the reduced regression model are described by a polynomial equation, composed of statistically significant coefficients:


Y = −0.1222 + 0.0093X_1_ + 0.1258X_2_ + 0.0001X_1_X_1_ + 0.0048X_3_X_3_ +0.0006X_1_X_2_ − 0.0194X_2_X_3_ (2)

**Figure 4:**
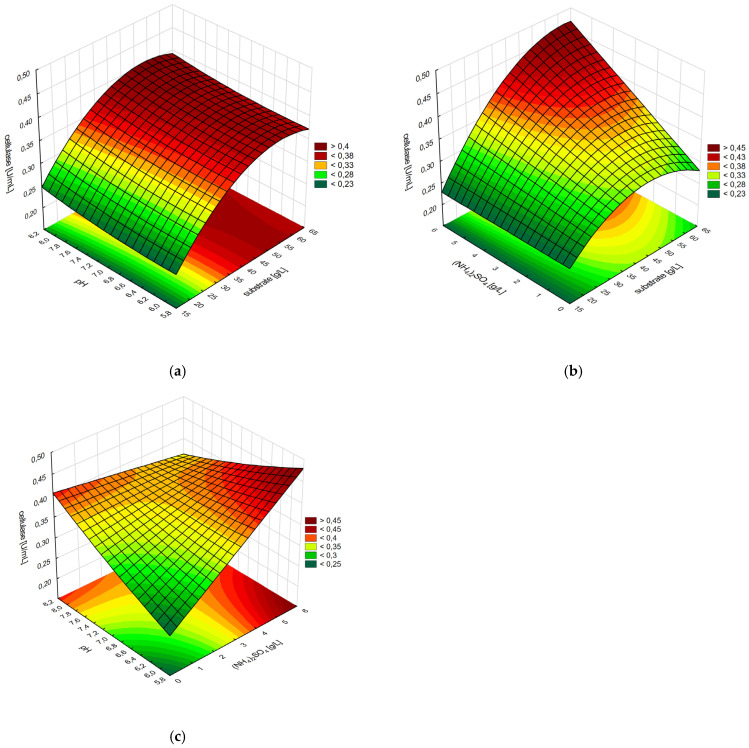
Response surface plots of interactive effects for pairwise sets of variables in the reduced model: (**a**) substrate concentration and medium pH; (**b**) concentration of substrate and ammonium sulfate; (**c**) ammonium sulfate concentration and medium pH.

The plotted response surfaces show the simultaneous influence of two independent variables and their interactions on the output variable. The curvature of the substrate concentration influence on the cellulase production resulted in the area in which the optimum substrate content could be established (Figure 4a). However, according to the interaction between the concentrations of substrate and ammonium sulfate, different curvature was observed at low and high levels of the ammonium sulfate dose (Figure 4b). From the interaction plot of ammonium sulfate and pH, the highest cellulase production was observed on the border of the experimental layout, at the highest level of the former (5.5 g/L) and lowest of the latter (pH 6.0) (Figure 4c).

Considering the confidence interval, the maximum predicted cellulase activity was 0.469 U/mL (0.434–0.505 U/mL) at 48.6 g/L substrate, 5.3 g/L ammonium sulfate, and pH 6.1. As a result of optimization, the cellulase activity was three times higher than its initial value.

The obtained value remains relatively low when compared to established strains or commercial cellulase. Higher cellulase activity of *B. subtilis* CD001 at the level of 1.85 U/mL was described by Malik and Javed [44]. However, the enzyme was produced on an easily degradable carbon source, CMC, in contrast to a natural, more difficult to hydrolyze substrate, BSG, used in our study. The authors concluded that natural carbon sources, i.e., sugarcane bagasse and wheat straw, to a lesser extent induced cellulase production (1.47 U/mL and 1.31 U/mL, respectively). Similar results were obtained by Sajitha et al. [45], who confirmed that CMC was a better cellulase inducer than natural substrates used in their study (banana flour, tapioca, potato, or banana peel). In turn, Moreu et al. [46] evaluated the use of cellulose from the primary pulp and paper industry sludge for the production of cellulases, but also hydrogen and ethanol, by *Clostridium thermocellum*. The maximum recorded cellulase activity reached the level of 0.25 U/mL and, despite relatively low enzymatic activity after 60 h of incubation, the cellulose substrate was completely hydrolyzed. For large-scale production of cellulolytic enzymes, soluble sugars or purified substrates are preferred. However, enzyme preparations produced in this way may lack the accessory enzymes required for biomass hydrolysis, as shown by Adsul et al. [47]. For this reason, it is recommended that microbial cellulases are produced either in the presence or solely on lignocellulosic raw materials, which tend to induce the production of valuable accessory enzymes that support hydrolysis of cellulose, e.g., pectinases [48], and/or non-hydrolytic proteins, i.e., the expansin-like protein [49]).

The initial pH of culture media is frequently regarded as one of the critical parameters during cellulase production processes with bacteria from the genus *Bacillus*, either when grown on CMC or natural lignocellulosic materials. Regardless of substrate type, optimization studies most often confirmed that a near-neutral to moderately alkaline pH (6.5–8.0) was most beneficial. However, some studies found the initial pH to be insignificant within a specific range [42,50,51,52,53]. Another principal parameter to be optimized is the concentration of the main substrate. When CMC is used as the main component, its optimal concentration is usually placed within the range 10–20 g/L [37,39,50,54,55]. As to natural substrates, a wide variety of examples is documented in the literature. Different agro-industrial by-products were evaluated in search for rational valorization of their biomass, and their concentration in liquid culture media was generally higher as compared to CMC-based media. Potato peel was used at concentrations of 20 g/L and 40 g/L in cellulase production with *B. aerius* MG597041 and *B. subtilis* K-18, respectively [52,56]. *B. subtilis* BM1 cultured on wheat bran required optimally 7.5 g/L of the substrate [51], while *B. subtilis* M1 cultured on groundnut shell required 20 g/L [57]. Pretreated sugarcane bagasse at optimal 50 g/L was used to culture *B. licheniformis* MTCC 429 [58], and coconut mesocarp waste at 40 g/L was suitable for *B. amyloliquefaciens* FW2 [59].

### 3.6. Raw BSG Hydrolysis with Concentrated Enzyme

In the process of enzymatic hydrolysis of BSG at 50 °C, after 20 h of reaction, the enzyme exhibited considerable thermostability, retaining 65% of the initial activity. The concentration of reducing sugars after the 20 h hydrolysis was three times higher than the initial concentration and was 2.6 g/L (130 mg/g; saccharification 8.5%). According to Yu and Li [60], in studies on the enzymatic hydrolysis of corn stover and rice straw using cellulase from *Gracilibacillus* sp. SK1, the obtained yield of reducing sugars from corn stover and rice straw was higher, 27.1 g/L (48 h) and 20.4 g/L (64 h), respectively, but required a much longer hydrolysis time. However, unlike our study, the cited experiments were conducted on cellulose substrates pretreated with NaOH (0.1 M) for 1 h at 120 °C, which significantly increases the availability of cellulose substrate to enzymes.

The hydrolytic efficiency of enzymatic preparations, including cellulase-containing cocktails, can be compared based on the criterion of the degree of cellulose conversion to fermentable sugars in the shortest possible time [47]. Efficient hydrolysis of natural lignocellulosic biomass requires the participation of numerous enzymes in different ratios, which is difficult to achieve with a wild-type microorganism. Therefore, synergistic enzyme cocktails are being developed as a new strategy to enhance biomass hydrolysis [61]. As an example, Takano and Hoshino [62] optimized a preparation containing three commercial enzymes for the hydrolysis of alkali-pretreated rice straw. The tailored mixture of “Cellulase Onozuka 3S”, “Cellulase T Amano 4”, and “Pectinase G Amano” improved the hydrolysis efficiency and its yield reached 94%.

### 3.7. Pretreated BSG Hydrolysis with Concentrated Enzyme

**Figure 5:**
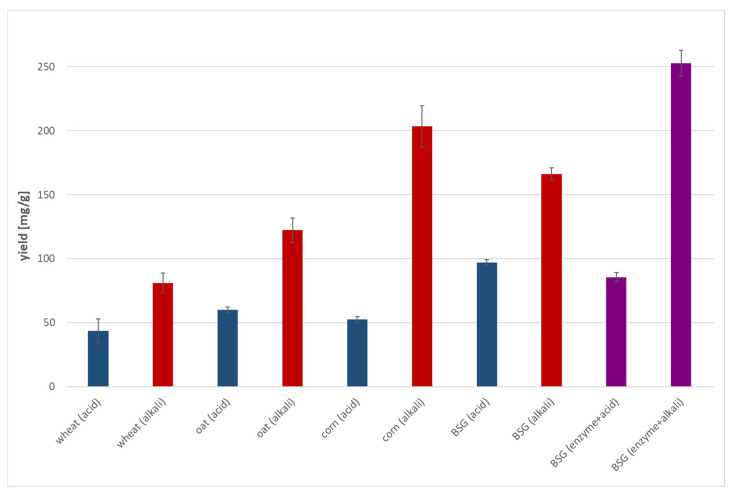
Yield of reducing sugars from enzymatic hydrolysis of both enzymatic and acid/alkali pretreated lignocellulosic materials, after 48 h of reaction.

Lignocellulosic substrates, BSG, wheat, oat, and corn, were subjected to two different pretreatments (acid and alkaline) before exposure to cellulase obtained from the tested strain. An alkaline pretreatment markedly accelerated the cellulose hydrolysis for all the lignocellulosic substrates tested (Figure 5). Lemões et al. [25] compared alkaline and acidic treatments of lignocellulose form *Arundo donax* L., finding the alkaline method to be more effective. Moreover, the authors highlighted additional advantages of alkaline treatment, including the elimination of acid neutralization and reduced water consumption for washing the treated biomass. In our study, additionally, the spent grain previously used for the production of cellulolytic enzymes was also subjected to either acidic or alkaline pretreatment, and further used for enzymatic hydrolysis. The results obtained in our study indicate that the BSG used for the production of enzymes can be reused in the saccharification process, following alkaline treatment (glucose yield 253 mg/g; saccharification 25%). However, the results obtained for the hydrolysis of spent grain subjected to enzymatic and alkaline pretreatment (saccharification 25%) indicate a significant loss of sugars due to the action of alkali.

### 3.8. Characterization of the Partially Purified Cellulase

Cellulase produced by *B. methylotrophicus* in the optimized BSG-based culture medium was concentrated with ultrafiltration and subjected to two-step column chromatography purification. As a result, partially purified cellulase was obtained that exhibited specific activity of 2.14 U/mg after IEC and 4.24 U/mg after GF chromatography, in contrast to 0.12 U/mg found in the raw culture fluid (Table S2). Zymographic analysis revealed a single activity band of approx. 70 kDa m.w. (Figure S1).

The partially purified cellulase exhibited activity in a broad range of pH, especially extended toward alkaline, with its maximum between pH 6.5 and 7.0. The optimal temperature oscillated between 50 °C and 60 °C (Figure S2). Among other producers of alkaline cellulases, bacteria from the genus *Bacillus* are frequent, e.g., *Bacillus* sp. DUSELR13 [63], *B. sphaericus* JS1 [64], and *Bacillus* sp. HSH-810 [65]. The optimum temperature for cellulolytic activity typically does not exceed 60 °C. Nevertheless, Azadian et al. [13] described a thermostable cellulase produced by *B. licheniformis* AMF-07, with an optimum temperature of 70 °C; however, strong enzyme inactivation occurred above this threshold. In turn, in terms of the temperature and pH profile, the *B. methylotrophicus* cellulase resembles the enzyme obtained in the culture of *Thermoanaerobacterium* sp. R63 described by Harnvoravongchai et al. [66]. The cellulase of this strain was thermostable with an optimal temperature at 65˚C, and exhibited activity in a broad range of pH, 5.0–9.0. The stability of cellulases at higher temperatures and within a wide range of pH is certainly an important asset in terms of facilitating enzymatic hydrolysis processes on an industrial scale. Thermostability of enzymes enables the application of higher process temperatures, facilitates the course of the hydrolysis process, and reduces the risk of contamination with mesophilic microflora [13].

Kinetic parameters of the tested cellulase were determined, i.e., K_m_ = 33.8 mg/mL and V_max_ = 135.1 μmol/min. The K_m_ and V_max_ values of CMC-ases reported in the literature for different microorganisms have a wide range and several-fold span, as inferred from available data in the BRENDA database [67]. In the case of *B. velezensis* A4, the reported K_m_ and V_max_ take values 63.4 mg/mL and 55.6 mg/min [68]. Different kinetic parameters were reported for *B. methylotrophicus* Y37, namely K_m_ = 0.19 mg/mL and V_max_ = 7.46 μmol/min [69].

The effect of inhibitors and surfactants on the partially purified cellulase activity was tested. The cellulase activity was clearly inhibited by cations of heavy metals, Zn^2+^, Cu^2+^, and Mn^2+^, which caused its reduction by 70 to 90% (Table S3). In turn, EDTA, which chelates divalent metal ions, reduced the activity by 30%, which may suggest the metal dependence of the enzyme. The presence of surfactant Triton X-100 in the reaction mixture resulted in a moderate increase in activity (124% residual activity). On the contrary, Tween 80 caused a slight reduction in activity, while Brij 35 appeared to have no major effect. A comparable effect of surfactants was demonstrated in the case of purified *B. licheniformis* AMF-07 cellulase in contact with detergents such as Dioxigene (122% residual activity) and Shooma (116% residual activity) [13]. The detergent-compatible cellulase preparations are commercially manufactured to meet the requirements of the process conditions [70].

### 3.9. Chemical Composition Analysis of Spent Grain

Ethanol-soluble substances in the sample of spent grains subjected to enzymatic hydrolysis were present in the amount of 21.09% of absolute dry weight (a.d.w.) (Table 3). The hot alkali solution extracts low molecular weight carbohydrates from wood and other lignocellulosic materials, mainly consisting of hemicelluloses and degraded cellulose. The cellulose content in the extracted sample of enzymatically hydrolyzed draff was 18.47% a.s.m. In turn, the product obtained by the method proposed by Klason [27], at an amount of 15.12% a.d.w., contained admixtures of polysaccharide decomposition products; furthermore, part of the methoxy groups were cleaved off during its preparation [71]. For comparison, the physical treatment of hardwood lignin with hot water, described by Ko et al. [17], showed that the content of acid-insoluble Klason lignin increased from 28.1% (for an untreated sample) to 37.6% (for a sample treated for 15 min at 220 °C in a fluidized sand bath). The results obtained indicate that more extractive substances, cellulose, Klason lignin, and pentosans (25.26% a.d.w.) were extracted from the sample of spent grains compared to the sample of raw grain, which confirms the effectiveness of hydrolysis with the participation of cellulolytic enzymes released by the *B. methylotrophicus* strain. Only the content of hemicelluloses in both samples was at a similar level of 74% a.d.w. The hot alkali solution used in this assay extracts low molecular weight carbohydrates from lignocellulosic materials, consisting mainly of hemicelluloses, although some cellulose and lignin also pass into the extract. Furthermore, pentosans are a component of hemicellulose; therefore, the percentages listed in Table 3 do not sum to 100%.

Shimizu et al. [72] reported an increased cellulose content by 5% after acidic pretreatment of banana pseudostem residue (H_2_SO_4_ 30% *w*/*w*, at 121 °C for 30 min). In our study, a similar effect was achieved using only an enzymatic treatment, where the cellulose content increased by 45%. This highlights the effectiveness of the enzymes in breaking down BSG to release more extractives, while also supporting the conclusion that chemical pretreatment of the substrate may be rendered redundant.

### 3.10. SEM Analysis of Raw and Enzymatically Treated BSG

**Figure 6:**
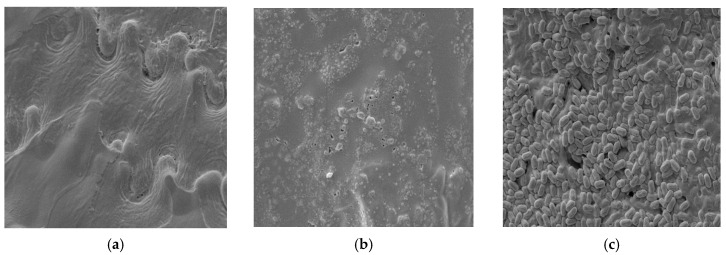
Morphological changes: (**a**) raw BSG; (**b**) enzymatically hydrolyzed BSG; (**c**) BSG after bacterial culture. Magnification ×4 k.

The morphological changes in spent grain as a result of enzymatic treatment were examined using SEM imaging (Figure 6), in relation to the untreated control. In microscopic images of spent grains subjected to enzymatic hydrolysis in contrast to raw spent grains, there is clear damage to the flat and smooth cellulose structure of barley husk (Figure 6a) caused by enzyme activity (Figure 6b). A similar effect can be observed on spent grain samples after bacterial treatment, where, in addition to the development of a bacterial biofilm, crack damage is also visible on the surface of the material (Figure 6c). Similar changes in the structure of cellulose were described by Ko et al. [17], where disruption and fragmentation of pretreated wood were demonstrated, in contrast to a smooth surface of untreated sample. However, unlike that study, the authors of the publication cited above subjected the cellulose material to pretreatment with the use of physical factors (hot water).

## 4. Conclusions

In this study, bacterium identified as *B. methylotrophicus* demonstrated potent cellulolytic abilities and rare resistance to high concentrations (70%) of ethanol and methanol. The combination of both traits is beneficial from the perspective of bioethanol production. The efficiency of the *B. methylotrophicus* to utilize selected lignocellulosic waste from the food industry for cellulase production was studied. The highest level of cellulase activity was obtained in the culture medium based on BSG, which was used in further research. The cellulase production conditions were optimized using a set of experimental designs. The maximum approximated cellulase activity was 0.469 U at 48.6 g/L substrate, 5.3 g/L ammonium sulfate, and pH 6.1. Alkaline pretreatment of waste cellulosic substrates proved to be more effective compared to acid treatment. The results indicate that more extractive substances, cellulose, Klason lignin, and pentosans (25.26% a.d.w.), were extracted from the sample of BSG compared to the sample of raw grain, which confirms the effectiveness of hydrolysis with the participation of cellulolytic enzymes produced by the *B. methylotrophicus* strain. SEM observations revealed the consequence of cellulase action and the biofilm formed by the tested strain. To our knowledge, this is the first report on *B. methylotrophicus* in utilizing untreated and pretreated BSG as a substrate for cellulase production, and indicates an alternative way for sustainable management of this by-product by utilizing it as a renewable, cheaper substrate for cellulase production that may serve in obtaining glucose for the bioethanol production.

## Figures and Tables

**Table 1:** Experimental layout according to Box–Behnken design with natural values of independent variables, and observed and predicted values of the response.

Run	Substrate [g/L]	(NH_4_)_2_SO_4_ [g/L]	pH	Cellulase [U] Observed	Cellulase [U] Predicted
1	20	0.5	7	0.265	0.259
2	60	0.5	7	0.268	0.312
3	20	5.5	7	0.291	0.275
4	60	5.5	7	0.422	0.455
5	20	3.0	6	0.246	0.263
6	60	3.0	6	0.403	0.379
7	20	3.0	8	0.276	0.281
8	60	3.0	8	0.451	0.397
9	40	0.5	6	0.308	0.274
10	40	5.5	6	0.469	0.451
11	40	0.5	8	0.393	0.389
12	40	5.5	8	0.370	0.372
13	40	3.0	7	0.342	0.367
14	40	3.0	7	0.344	0.367
15	40	3.0	7	0.362	0.367

**Table 2:** ANOVA table for the reduced regression model: R^2^ = 0.8999, adj. R^2^ = 0.8248.

Model Component	SS	df	MS	F	*p*
substrate [g/L] (L)	0.0272	1	0.0272	217.13	0.0046
substrate [g/L] (Q)	0.0058	1	0.0058	46.46	0.0209
(NH_4_)_2_SO_4_ [g/L] (L)	0.0126	1	0.0126	100.54	0.0098
pH (Q)	0.0042	1	0.0042	33.73	0.0284
1 L × 2 L	0.0041	1	0.0041	32.75	0.0292
2 L × 3 L	0.0085	1	0.0085	67.93	0.0144
lack of fit	0.0068	6	0.0011	9.02	0.1031
pure error	0.0003	2	0.0001		
total SS	0.0702	14			

**Table 3:** Analysis of the chemical composition of spent grain in terms of the content of cellulose, lignin, and hemicellulose.

Parameter	Raw BSG * % a.d.w	Hydrolyzed BSG % a.d.w
Moisture	4.66 ± 0.14	5.94 ± 0.08
Moisture after ethanol extraction	7.14 ± 0.16	6.48 ± 0.13
Extractives	14.24 ± 0.21	21.09 ± 0.13
Cellulose	12.74 ± 0.06	18.47 ± 0.16
Klason lignin	10.25 ± 0.68	15.12 ± 0.04
Hemicellulose	74.35 ± 0.13	74.24 ± 0.24
Pentosans	24.79 ± 0.08	25.26 ± 0.03

* The analysis of the chemical composition of raw spent grain comes from previous research conducted in 2019 as part of the project 337015/N.

## Data Availability

The original contributions presented in this study are included in the article/Supplementary Material. Further inquiries can be directed to the corresponding author.

## Supplementary Materials

The following supporting information can be downloaded at: https://www.mdpi.com/article/10.3390/ma18143256/s1. Figure S1: CMC-zymogram of partially purified cellulase from *B. methylotrophicus*; a—cellulase, b—protein ladder (PageRuler Plus, Thermo Scientific). Figure S2: The effect of pH (a) and temperature (b) on the cellulase activity (circles—acetic buffer, squares—phosphate buffer). Table S1: Experimental layout of the Plackett–Burman screening design with natural levels of independent variables, along with the observed values of the response. Table S2: Purification table of cellulase from *B. methylotrophicus*. Table S3: The effect of different compounds on cellulase activity. Reference [73] is cited in the Supplementary Materials.

## Abbreviations

The following abbreviations are used in this manuscript:

BSG	Brewer’s spent grain
CMC	Carboxymethyl cellulose
DNS	3,5-Dinitrosalicylic acid
YE	Yeast extract
PCR	Polymerase chain reaction
SEM	Scanning electron microscopy
TFF	Tangential flow filtration
