# Supplementary material for: Cellulolytic Potential of Newly Isolated Alcohol-Tolerant Bacillus methylotrophicus
Source: Materials (Basel). 2025 Jul 10;18(14):3256. doi: 10.3390/ma18143256 (PMC12300512; doi:10.3390/ma18143256)
Supplement: Supplementary file 1 [file materials-18-03256-s001.zip › materials-3734505-supplementary.pdf]

Table S1. Experimental layout of the Plackett-Burman screening design with natural levels of independent variables, along with the observed values of the response

run	substrate [g/L]	pH [g/L]	peptone [g/L]	YE [g/L]	$(NH_4)_2SO_4$ [g/L]	KNO_3 [g/L]	NH_4Cl [g/L]	cellulase act. [U] observed
1	15	6	0.0	1.0	1.0	1.0	0.0	0.192±0.024
2	40	6	0.0	0.0	0.0	1.0	1.0	0.255±0.033
3	15	7	0.0	0.0	1.0	0.0	1.0	0.253±0.022
4	40	7	0.0	1.0	0.0	0.0	0.0	0.257±0.018
5	15	6	1.0	1.0	0.0	0.0	1.0	0.203±0.014
6	40	6	1.0	0.0	1.0	0.0	0.0	0.299±0.043
7	15	7	1.0	0.0	0.0	1.0	0.0	0.215±0.040
8	40	7	1.0	1.0	1.0	1.0	1.0	0.337±0.027

Table S2. Purification table of cellulase from *B. methylotrophicus*

	volume [mL]	activity [U]	protein [mg/mL]	specific activity [U/mg]	total protein [mg]	yield [%]	purification fold
culture fluid	500	0.562	4.877	0.115	281	100.0	1.0
ultrafiltration	120	1.691	1.7	0.995	202.92	72.2	8.6
HiTrap DEAE	15	1.188	0.555	2.141	17.82	6.3	18.6
HiPrep Sephacryl S-200	5	3.221	0.76	4.238	16.105	5.7	36.8

Table S3. The effect of different compounds on cellulase activity

Compound	Residual activity [%]
$ZnSO_4$	8.9
$FeSO_4$	90.1
$MgSO_4$	97.7
$CuSO_4$	9.9
$CaCl_2$	89.9
$MnSO_4$	31.8
EDTA	69.8
Tween 80	86.8
Triton X-100	124.2
Brij 35	106.1

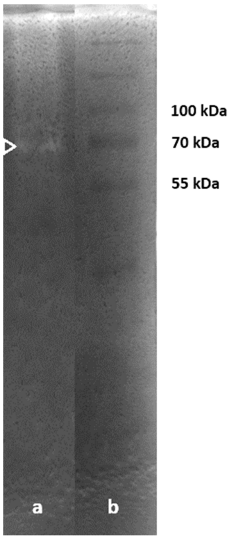

Figure S1. CMC-zymogram[1] of partially purified cellulase from *B. methylotrophicus*; a – cellulase, b - protein ladder (PageRuler Plus, Thermo Scientific)

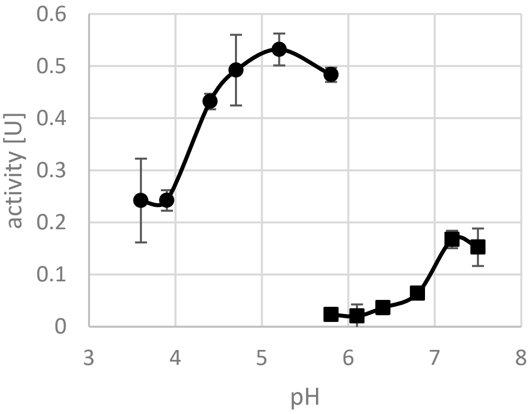

(a)

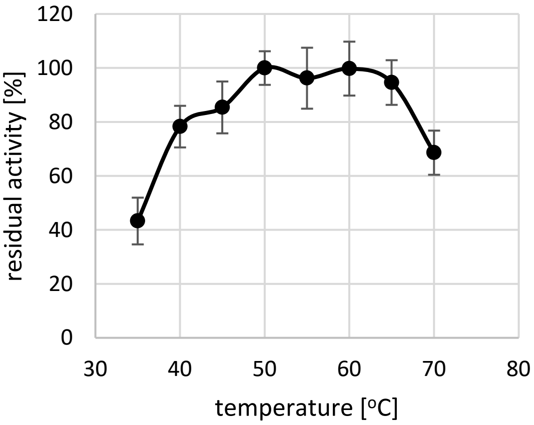

(b)

Figure S2. The effect of pH (a) and temperature (b) on the cellulase activity (circles – acetic buffer, squares – phosphate buffer).

[1] performed according to: Potprommanee L, Wang X-Q, Han Y-J, Nyobe D, Peng Y-P, Huang Q, et al. (2017) Characterization of a thermophilic cellulase from *Geobacillus* sp. HTA426, an efficient cellulase- producer on alkali pretreated of lignocellulosic biomass. PLoS ONE 12(4): e0175004.
